# Implementation of a national anti-tuberculosis drug resistance survey in Tanzania

**DOI:** 10.1186/1471-2458-8-427

**Published:** 2008-12-30

**Authors:** Timothy M Chonde, Basra Doulla, Frank van Leth, Sayoki GM Mfinanga, Nyagosya Range, Fred Lwilla, Saidi M Mfaume, Armand van Deun, Matteo Zignol, Frank G Cobelens, Saidi M Egwaga

**Affiliations:** 1 National Tuberculosis and Leprosy Control Program, Ministry of Health and Social Welfare, Dar es Salaam, Tanzania; 2 KNCV Tuberculosis Foundation, The Hague, the Netherlands; 3 Center for Infection and Immunity Amsterdam (CINIMA), Academic Medical Center, University of Amsterdam, the Netherlands; 4 National Institute for Medical Research, Muhimbili Medical Research Centre, Dar es Salaam, Tanzania; 5 Mycobacteriology unit, Institute of Tropical Medicine, Antwerp, Belgium; 6 Stop TB Department, World Health Organization, Geneva, Switzerland

## Abstract

**Background:**

A drug resistance survey is an essential public health management tool for evaluating and improving the performance of National Tuberculosis control programmes. The current manuscript describes the implementation of the first national drug resistance survey in Tanzania.

**Methods:**

Description of the implementation process of a national anti-tuberculosis drug resistance survey in Tanzania, in relation to the study protocol and Standard Operating Procedures.

**Results:**

Factors contributing positively to the implementation of the survey were a continuous commitment of the key stakeholders, the existence of a well organized National Tuberculosis Programme, and a detailed design of cluster-specific arrangements for rapid sputum transportation. Factors contributing negatively to the implementation were a long delay between training and actual survey activities, limited monitoring of activities, and an unclear design of the data capture forms leading to difficulties in form-filling.

**Conclusion:**

Careful preparation of the survey, timing of planned activities, a strong emphasis on data capture tools and data management, and timely supervision are essential for a proper implementation of a national drug resistance survey.

## Background

Drug resistance can occur due to inadequate management of treatment or transmission of drug resistant strains. Multi-drug resistant tuberculosis (MDR-TB) is defined as tuberculosis (TB) resistant to isoniazid and rifampicin, the two main first-line drugs in the treatment of TB. In 2006, nearly 500,000 new MDR-TB patients were identified worldwide[1]. Treatment of these patients is feasible but is substantially more costly than treating patients with fully susceptible *Mycobacterium Tuberculosis *strains[2].

The growing prevalence of MDR and the emerging threat of XDR-TB (MDR-TB with additional resistance to at least any of the fluroquinolones and one of the three injectable second-line TB drugs) urged the World Health Organization (WHO) to form a global task force on MDR/XDR-TB [3], which charged the Working Group on MDR-TB of the STOP TB Partnership to develop a response plan to address this emergence[4]. In this, countries are advised to perform drug resistance surveys to be able to provide insight in the magnitude of the resistance problem. Given the underlying causes of drug resistance, information on TB drug resistance levels is an essential public health management tool for evaluating and improving the performance of the National TB control Programmes (NTPs), and indicates the difficulties which NTPs will encounter when administering chemotherapy[1,3,4].

For the United Republic of Tanzania, information on TB-drug resistance is available from studies covering specific districts or diagnostic facilities[5], in the context of TB-HIV studies[6,7], and from baseline information of patients enrolling in a randomised clinical trial[8]. All of these studies showed low levels of drug resistance and MDR. The routine data analysed from the Central Tuberculosis Reference Laboratory (CTRL) also showed low levels of drug resistance of *Mycobacterium tuberculosis *strains tested from 1991 to 1999. Rates were 10% to any drug, 5.6% to isoniazid, 0.5% to rifampicin, 0.6% to ethambutol, 1.4% to streptomycin and around 1% to isoniazid and rifampicin (MDR-TB).

The data of these studies and of the routinely collected specimens are not adequately informative on the prevalence of drug resistance on a national level, because specimens were not collected from a representative sample of TB patients throughout the country, and the surveys were not in accordance with current internationally recommended methodology[9]. The only national surveys on drug resistance were carried out before or just after the introduction of current TB-control strategies [10-12]. For this reason, the National Tuberculosis and Leprosy Programme (NTLP) of Tanzania has implemented for the first time a drug resistance survey (DRS) on a national representative sample of TB patients identified in the country.

The current manuscript describes the methodology used, the logistics involved, and the factors contributing to its implementation. The positive and negative lessons learned from the implementation of the survey can be used by other countries that are embarking on a similar survey.

## Methods

### Study population and sample size

The DRS is implemented in 40 diagnostic centres (denoted as clusters) which were sampled proportional to patient load (new smear-positive cases) from all diagnostic centres notifying TB cases to the NTLP. Each cluster enrolled 30 consecutive new smear-positive TB cases, arriving at a self-weighting study sample. During the enrolment time, the clusters were required to enrol consecutively all re-treatment cases diagnosed. Inclusion closed after 12 months even if enrolment of required cases was not complete.

### Inclusion criteria

All sputum-positive patients diagnosed during the inclusion period were eligible to be enrolled in the study. Smear-positivity was diagnosed according to the national guidelines requiring at least two out of three sputum samples showing acid-fast bacilli by Ziehl Neelsen (ZN) microscopy. A re-treatment case was defined as a patient with smear-positive TB who received treatment for TB for more than one month. All other patients were classified as new.

### Sample collection and processing

All diagnosed patients provided a new morning sputum specimen the next day. This specimen was sent to CTRL without interfering with. If the expected transit time between cluster and CTRL was assessed to take longer than 4 days, two morning specimens were collected; one with Cetyl-Pyridinium-Chloride (CPC) and one without CPC added for preservation purposes[13,14]. All specimens were examined by ZN microscopy and culture. Culture was performed on solid Loewenstein Jensen medium after decontamination by modified Petroff's method. The results of the sensitivity tests were interpreted with the proportion method[15].

The study was approved by the National Ethical Committee which has its secretariat at National Institute for Medical Research.

## Results

### Initiation of the study

**Figure 1:**
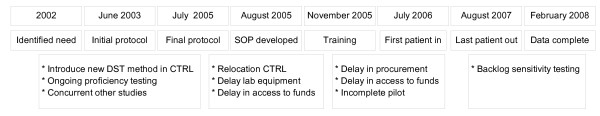
**Time line and constraints drug resistance survey Tanzania**.

The initial discussions of implementing the DRS started as early as 2002 amongst the major stakeholders including NTLP, WHO, Institute of Tropical Medicine in Antwerp, KNCV Tuberculosis Foundation in The Netherlands, and the National Institute for Medical Research, Muhimbily Centre in Tanzania. Figure 1 shows the timeline of the DRS in Tanzania. It can be seen that factors related to infrastructure, personnel, finance, and time all played a crucial role in the long duration of the DRS from initiation to analysis.

From an initial budget of 20,000 USD, the study costs increased up to 100,000 USD at the actual start of the study due to increased costs of personnel, transport arrangements for specimens, and supplies. The continuous commitment of the stakeholders towards the study and the willingness to meet the rising financial demands proved to be instrumental in the final implementation of the study 4 years later.

### Preparation of the study

An essential step in the successful implementation was the pre-survey visit to each selected cluster by a laboratory-experienced member of the study team. The cluster was assessed using a pre-defined checklist for personnel, equipment, and infrastructure. The biggest gain of these visits was that a detailed and novel cluster-specific plan for transporting the samples to CTRL was made.

Special contracts with bus companies were negotiated that agreed on delivery of the samples and the costs involved. Because bus companies were paid upfront, there was no need to negotiate charges when the samples were sent. Staff of the clusters was given mobile phone top-up cards to be able to call CTRL to inform that samples were put on the bus. CTRL staff then went to the delivery point for pick-up. For clusters close to CTRL, 'specimen boys' were hired who's sole task was to collect samples at the clusters and bring them to CTRL in person. These individuals were paid per quarter assuring their continuous commitment to the job. Transport containers and CPC were provided for by the programme and distributed prior to the study.

The organization of the travel arrangements took several months to complete, but paid off. In routine settings, the specimens from the remote areas would have been sent by mail and take up to several weeks to arrive at CTRL. During the DRS, the median transport time of all samples was reduced to only 5 days and stayed this low during the entire survey. This includes specimens from remote areas. Consequently, the yield of the culture was above 90%. This could not have been achieved without paying much detail to the logistic involved in sample transportation.

### Training of study personnel

The training of all the study personnel took place at six different places and was organised by the staff from CTRL. In the two-day event, the participants were informed on the rationale for the study, the protocol, and the data capture forms. The participants engaged in a practical exercise on interview-taking and form-filling.

An important factor negatively influencing the implementation of the DRS was the long time between the training and the actual start of the survey. For some clusters, this delay ran up to 9 months. In that time, especially administrative issues related to the form-filling were often forgotten. In all clusters, at least one, but sometimes all, trained staff member was replaced between training and start of survey activities in the cluster. The budget did not foresee training of the replacement staff. Instead, new staff was trained on-the-spot by laboratory personnel who had received the initial training. This situation resulted in regularly observed incomplete data capture forms with internally inconsistent data. This complicated data management and data validation procedures, which increased the workload of CTRL staff.

### Implementation of the study

All clusters performed a pilot study in four TB-suspects to assess the study procedures and the transport of specimens. No major issues came to light during the pilot study, although the actual processing of the specimens and the data recording were not part of the test. This shows that results from pilot studies are only useful if no major changes in the study activities occur. The change in research staff during the survey made it impossible to meet this prerequisite.

The factor that had the largest influence on the implementation of the study was the design of the data capture forms, which were not formally pre-tested. This resulted in frequently observed incomplete data capture forms, especially regarding study number and type of patient (new or retreatment). To be able to monitor the inclusion in the study, all forms were recoded manually from the midpoint of the survey onwards. This identified 13 (32%) clusters that had stopped submitting samples before reaching the target enrolment. Thirty-five (3%) extra samples were received afterwards from these clusters.

The final classification on the type of patient was absent for 292 (19.2%) of the 1520 data capture forms. Of these, 85 had no interview data recorded at all. The status of these patient was derived by contacting the cluster. All remaining forms with incomplete interview data (n = 207) were assessed manually at the end of the study to classify the type of patient.

Apart from these two critical variables, age was not recorded on 42 (3%) forms, sex not on 5 (0.3%), and date of specimen not on 54 (3.6%).

The problems with the form-filling could not be solved during the survey because the largest clusters recruited their patients in a very short time frame. This made monitoring visits being too late. Monitoring visits that were planned for the other clusters were not implemented fully due to lack of personnel and difficulties in access to funds. As an alternative, a statistical syntax was written that could identify missing data and internal inconsistencies. This syntax was run on updates from the database at regular intervals. The identified database records with errors were manually checked against the data capture forms and corrected where needed. Issues that could not be solved by this procedure were corrected during routine supervision visits to the clusters by the NTLP.

These negative influences need to be anticipated by countries that are embarking on a similar survey. There can be not enough emphasis on pre-testing of data capture forms and frequent monitoring of data collection. Post-hoc corrections of errors and inconsistencies will delay the reporting of the results and the use of the results by the NTLP for planning purposes.

The processing of the specimens during the study was without major technical problems. The progress was, however, hampered by the unexpected lack of equipment, leading to a constant backlog in sensitivity testing on already performed cultures. The ability to carry out frequent sub-culturing assured that no samples were lost for the survey, but added to the workload of the laboratory personnel. The delay in sensitivity testing caused that patients and their treatment providers were not informed on the presence of drug resistance, what can be seen as missed opportunity to contribute to individual patient care. Also, the delay in sensitivity testing precluded early guidance from the supranational reference laboratory (SNRL) in Antwerp on the DST technique. Despite this, the ongoing proficiency testing by the SNRL showed a high level of accuracy of the susceptibility testing of the samples from routine surveillance. In the latest round of testing, the efficiency (ratio between correct results and total results) of CTRL was 100% for isoniazid, 97% for rifampicin, 93% for streptomycin, and 100% for ethambutol. Since this technique is exactly the same as the technique used in the survey, the quality of the DST results in the survey is assured.

### Data management

All information needed for the study was documented in study-specific registers at CTRL. The main challenge was to transfer the information from the registers to the data capture forms and making them available for data entry. The data manager of the NTLP was not involved in the study due to other activities. No replacement was employed. Therefore, monitoring of data management was only performed during visits of a technical advisor from abroad. This is an undesirable situation because it delays assessment of the data, precludes adjusting data entry errors, and lacks capacity building activities within the NTLP. Countries that will embark on a DRS should invest in proper data entry and data management personnel that can perform the activities in real-time.

## Discussion

The initiation, preparation and implementation of the DRS turned out to be challenging. The whole process revealed some valuable lessons to be learned that are summarized in Table 1.

**Table 1:** Recommendations for implementing a drug resistance survey

Ensure ongoing commitment for the study from key stakeholders
Train personnel only when the study can start directly after the training
Prepare detailed SOPs at the laboratory including recording procedures
Make cluster-specific arrangements for sample shipmen
Ensure enough personnel to perform monitoring visits right from the start
Ensure availability of all required laboratory equipment in relation to specimen load.
Pre-test data capture forms
Train or retrain data entry clerks in study-specific procedures
Involve an experienced data manager for ongoing data management
Ensure early and regular transport of strains for rechecking to the supra-national laboratory
Ensure collaboration with a research institutions to adequately make use of available resources

Ensuring the ongoing commitment for the study from key stakeholders proofed to be vital. Without this commitment there would have been no drive to address the problems identified before and during implementation. It assures that the final data will be used for planning purposes of the NTLP.

The issues that influenced the conduct of the survey negatively are well known in the field of project management. It remains surprising to see how large the impact of these issues is on the efficient implementation of a study. Most national tuberculosis programmes do not have the luxury of being able to free personnel solely for research purposes, making it difficult to perform efficient research. However, engaging research scientists from research institutions that are able to give 100% person time for the project could be an alternative solution and backup to the program. This can work very well provided upfront agreement and commitment in collaboration is secured.

The DRS in Tanzania showed that detailed design of data capture tools, investment in data entry personnel, data management personnel, and frequent monitoring visits can avoid many of the problems encountered in the present survey. In any DRS where sampling is performed proportional to patient load, there will be clusters that finish quickly with enrolment. These clusters should be the primary focus of the initial monitoring. Not only will this ensure valid data from these clusters, it will also guide the monitoring in other clusters on issues that only arise in a real-life situation rather than in a pilot study.

## Conclusion

The design and implementation of the drug resistance survey in Tanzania was a large undertaking which needed continuous commitment, adequate funding, enough personnel and equipment. Although well known project management issues were addressed in the design phase of the study, these still influenced the actual implementation at each stage of the project. Only with additional data validation measures was it possible to arrive at a valid data base that can be used for answering the research questions. Although NTP managers who are embarking on such a survey will have country-specific issues to solve, the lessons learned during the survey in Tanzania could provide precious guidance and facilitate the smooth implementation of such an important activity.

## Abbreviations

CPC: Cetyl-Pyridinium Chloride; CTRL: Central Tuberculosis Reference Laboratory; DRS: Drug Resistance Survey; HIV: Human Immunodeficiency Virus; MDR: Multi-Drug Resistance; NT(L)P: National Tuberculosis (and Leprosy) Programme; SNRL: Supra-National Reference Laboratory; SOP: Standard Operating Procedure; TB: Tuberculosis; USD: American Dollar; WHO: World Health Organization; XDR: Extensive Drug Resistance.

## Competing interests

The authors declare that they have no competing interests.

## Authors' contributions

TC and BD were involved in the design, implementation, and analysis of the survey, and provided critical comments the manuscript. FvL was involved in the design of the study, performed data validation and analysis, and wrote the first and all subsequent drafts of the manuscript. SMf, MZ, FC, and SE were involved in the design of the study and provided critical comments to the manuscript. NR, and FL were involved in the design and implementation of the survey, and provided critical comments to the manuscript. SMa was involved in the implementation of the survey and provided critical comments to the manuscript. AvD was involved in the design of the study, performed data validation and analysis, and provided critical comments to the manuscript.

## Pre-publication history

The pre-publication history for this paper can be accessed here:


